# Gastric Signet Ring Cell Carcinoma Metastasis From Lobular Breast Cancer: A Diagnostic Pitfall

**DOI:** 10.7759/cureus.59919

**Published:** 2024-05-08

**Authors:** Jessica Kartotaroeno, Caroline Bastid, Giacomo Puppa

**Affiliations:** 1 Division of Clinical Pathology and Forensic Medicine, Raymond Poincaré University Hospital, Garches, FRA; 2 Division of Gastroenterology and Hepatology, University Hospital of Geneva, Geneva, CHE; 3 Division of Clinical Pathology, University Hospital of Geneva, Geneva, CHE

**Keywords:** signet-ring cell carcinoma, rare entity, diagnostic pitfall, primary gastric carcinoma, invasive lobular carcinoma, gastric metastasis

## Abstract

Breast cancer is the most common type of cancer among women worldwide. Gastric metastasis from invasive lobular carcinoma of the breast is unusual.

We report the case of a 66-year-old woman, under follow-up for an invasive classic lobular carcinoma of the left breast treated four years prior, who was admitted for upper abdominal discomfort and worsening constipation. Linitis plastica was suspected at gastroscopy.

Histology of gastric biopsies showed a poorly cohesive carcinoma comprising signet ring cells, with no resemblance to the original breast cancer. An adequate immunochemistry panel, including estrogen receptor and GATA-3, eliminated primary gastric cancer and proved that the gastric lesion was a metastasis of the previously diagnosed invasive lobular breast cancer with additional signet ring cell differentiation, which is classified among its rare variants.

This challenging case shows the importance of oncologic medical history and immunochemistry in differentiating between metastasis from invasive lobular breast carcinoma and primary gastric cancer. The distinction is necessary as the prognosis and approaches for treatment are different.

When encountering a gastric signet ring cell carcinoma, one must keep in mind that it actually can be a metastasis from one of the several primary sites of origin.

## Introduction

Breast cancer is the most common type of cancer among women worldwide, particularly in industrialized countries, and the leading cause of female cancer death [1]. Bone, lung, liver, and brain are the main sites of metastasis. The gastrointestinal site is relatively uncommon, with an incidence ranging from 0.2 to 1.7% [2]. Although metastasis of breast cancer to the gastrointestinal (GI) tract is rare, when it does occur, the stomach is usually the most frequent site. It also presents as diffuse linitis plastica-like infiltration, similar to primary gastric cancer. Gastric metastasis from invasive lobular carcinoma (ILC) of the breast is reported to occur at a higher rate than the ductal subtype [3].

Signet ring cell carcinoma (SRCC) is a rare poorly differentiated aggressive subtype of adenocarcinoma that most commonly arises from the GI tract (in particular the stomach) and breast but every organ is a potential primary site [4,5]. At histology, it is a special type of poorly differentiated mucinous adenocarcinoma in which the nucleus is pushed to the periphery by the abundant intracytoplasmic mucin, conferring the similarity to a signet ring.

Besides the classic histotypes of breast cancer, namely ductal and lobular type, there are several variants reported, for the lobular the pleomorphic, solid, alveolar, and signet-ring type being included [6]. These rare variants when metastatic can be extremely difficult to differentiate from a primary tumor.

Despite its declining incidence, gastric cancer has the third highest cancer-related mortality rate [7]. The main histological subtypes of gastric cancer include intestinal (45%) and poorly cohesive including SRCC (20-54%) [7].

In this study, we report a case of gastric metastasis from classic invasive lobular breast cancer, occurring four years after initial diagnosis and presented as poorly cohesive carcinoma with signet ring cells, thus mimicking a primary gastric cancer. Breast cancer metastasis to gastric usually occurs 5-7 years post primary diagnosis [8]. The clinical presentation suggested primary gastric cancer due to the linitis plastica apparent in the endoscopy.

 It is this change in morphology from one type of ILC to another different histotype that is discussed in this article.

This constitutes a major diagnostic pitfall and only a detailed medical history and a pertinent immunochemical panel confirm the correct diagnosis. The identification of metastatic ILC has crucial consequences with regard to patient management and prognosis in contrast to primary gastric cancer because the indications for surgery and the chemotherapic regimens are different.

## Case presentation

A 66-year-old woman was under follow-up for breast ILC. The treatment included neo-adjuvant surgery by left mastectomy with axillary lymph node dissection. Surgery was followed by three cycles of chemotherapy. 

Four years later she was admitted to the University Hospital in Geneva for upper abdominal discomfort and worsening constipation, which had been progressing for five months.

**Figure 1:**
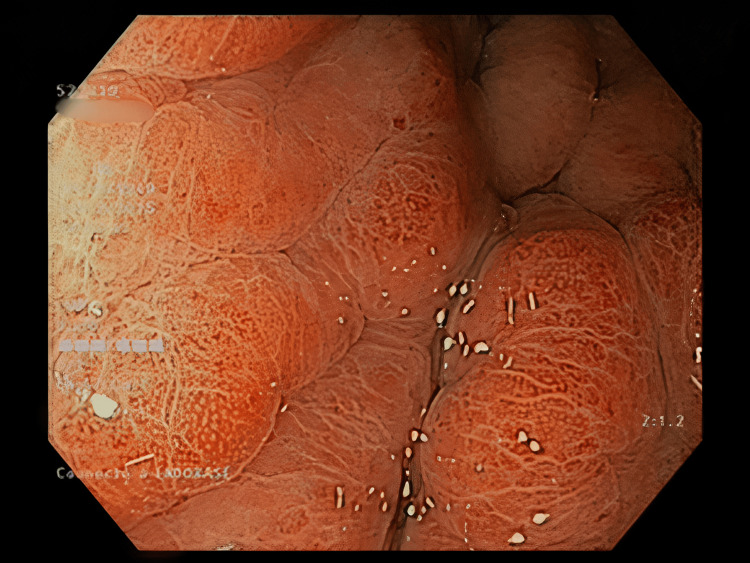
Gastroscopy Giant gastric folds.

**Figure 2:**
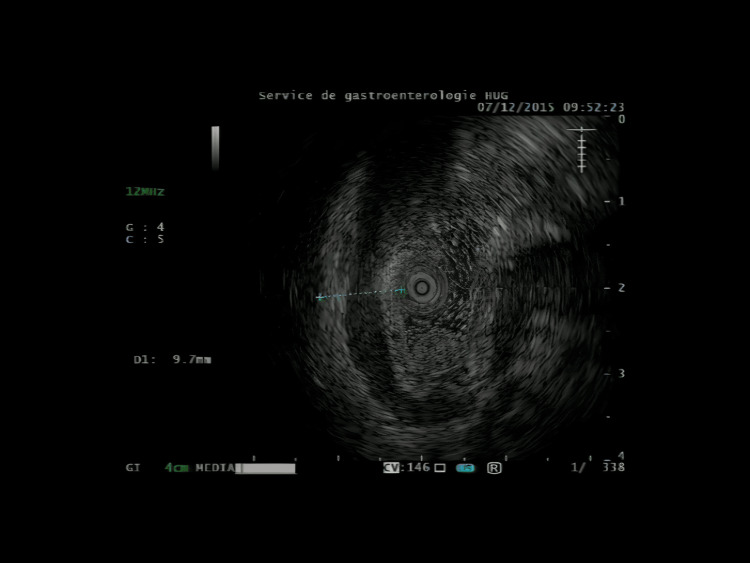
Gastric echoendoscopy Thickening of gastric wall.

The work-up started with a computed tomography scan which showed a diffuse thickening of the gastric wall, sparse infiltration of the colon, and peritoneum cavity. It also detected several suspicious metastatic vertebral lesions. She then underwent gastroscopy with echoendoscopy, which showed thick fundic gastric folds with high suspicion of a linitis plastica (Figures 1, 2). Biopsies showed gastric infiltration by SRCC.

Over the course of two years, the disease further evolved with metastases in the right orbital cavity invading the lachrymal gland which was treated with radiotherapy. Unfortunately, the patient died shortly after this multimetastatic disease.

Pathology analysis

Primary Breast Tumor

Macroscopic evaluation of the left mastectomy found a white, star-shaped and indurated mass, measuring 4.5 cm at its largest diameter, located behind the nipple.

**Figure 3:**
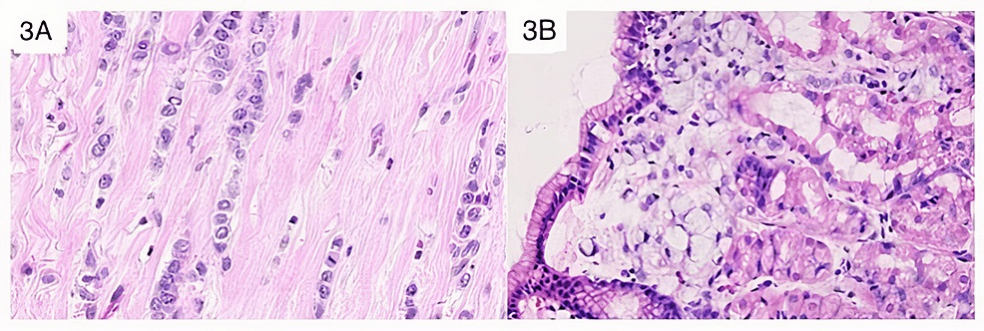
Comparative histology between breast and gastric lesions A: Breast tumor (previous mastectomy): classic invasive lobular carcinoma characterized by tumor cells arranged in single files, cords and single cells. No signet ring cell (hematoxylin and eosin staining x400). B: Gastric fundic biopsies: superficial clusters of tumoral poorly cohesive carcinoma- SRCC in the mucosa. Neoplastic cells are characterized by a central, optically clear, cytoplasmic mucin with an eccentrically placed nucleus (hematoxylin and eosin staining x400). SRCC: Signet ring cell carcinoma

Histology showed a larger lesion reaching 6.5 cm on its long axis and an infiltration of the deep margin, after an extensive sampling. All samples taken showed single files and cords of dyscohesive small, monomorphic cells, round or notched ovoid nuclei, in a desmoplasic stroma. These features were consistent with an invasive lobular breast carcinoma classic type according to World Health Organisation (WHO) (Figure 3A). Metastatic deposits were found in 8 over 10 axillaries lymph nodes thus making a TNM-VIII stage ypT3 N2a G2 R1. We have made a morphological comparison with the gastric recent lesion (Figure 3B).

The majority of cancer cells were strongly positive for estrogen receptor (ER) and progesterone receptor (PR). Mib1 was evaluated at 20%. The human epidermal growth factor receptor 2 (HER2) was non-amplified.

Gastric Biopsies

At histology, fundic gastric mucosa showed a poorly cohesive carcinoma (according to the WHO) with signet ring cell phenotype. The typical appearance was of cells characterized by a central, optically clear, cytoplasmic mucin with an eccentrically placed nucleus. The neoplastic single cells were present as scattered individual cells or clusters (Figure 3B).

**Figure 4:**
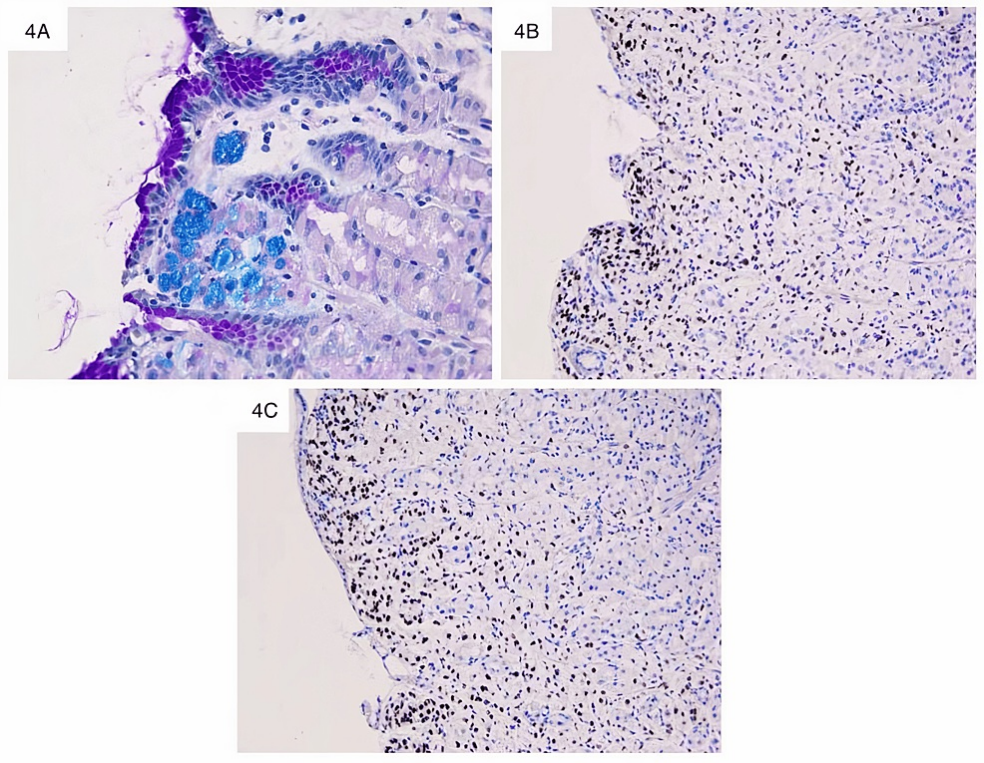
Gastric lesion with special stain and immunochemistry A: Intra-cellular mucin in vacuoles is Alcian blue-positive (Alcian blue staining x400). B: Isolated cells and tumoral clusters are ER-positive (ER immunochemistry x200). C: Nuclear GATA3-positivity in tumoral cells of gastric mucosae (GATA3 immunochemistry x200).

Special stain Alcian-blue highlighted the intra-cellular mucin seen in the signet ring cells (Figure 4A), without extra-cellular mucin thus confirming the morphological aspect. The signet ring differentiation enhanced by the special stain, usually encountered in primary gastric cancer, was completely different at morphology from the lobular variant of the resected breast tumor in the past. Keeping in mind the patient’s clinical history, we challenged the presentation and the suspicion of a primary gastric cancer, and we applied an immunohistochemical panel to distinguish it from metastatic breast cancer. Neoplastic cells were diffusely and strongly positive for pan-Cytokeratines (pan-CK), and breast marker differentiation as ER (Figure 4B) and GATA3 binding protein (GATA3) (Figure 4C). PR, not shown, was negative. Mib1 was evaluated at 15% and HER2 was not amplified.

This immunochemistry panel allowed to exclude a primary gastric cancer (typically pan-CK positive, ER and GATA3 negative) and to make the final diagnosis of a gastric metastasis from previously diagnosed invasive lobular breast cancer, with an additional signet ring cell differentiation, after comparison with the primary tumor resected four years prior.

## Discussion

Breast cancer is the most frequent tumor among women worldwide [1]. The more common histology subtypes are ductal (60-80%) and lobular (5-15%) [9].

Besides the classic lobular type, there are several variants reported, including the pleomorphic, solid, alveolar, and signet-ring cell types [6]. While 90% of signet-ring tumors arise from the stomach, breast, or colon, almost every organ is a potential primary site [4,5].

A signet ring cell-rich ILC of the breast was described in 1976 by Steinbrecher and Silverberger [10]. These pathologists characterized this entity with foci of >50 signet ring cells per high power field. It is a distinct clinicopathology variant of lobular carcinoma, defined as more than 20% of the malignant cells appearing as signet rings formed by mucin-positive intracytoplasmic vacuoles [10]. The signet ring variant of ILC is reported as 2 to 4.5% of all breast carcinomas [11].

In surgical series, breast cancer represented 8% of secondary metastatic malignancies of the GI tract [12]. Gastrointestinal metastasis in patients with lobular breast carcinoma usually manifests 5-7 years after the initial diagnosis [8]. ILC has a greater tendency to metastasize to the peritoneum, retroperitoneum, and gastrointestinal tract as compared to ductal carcinoma [13]. When an ILC spreads to the stomach, distinguishing such localization from a primary gastric cancer is extremely challenging, in particular when there is evidence of signet ring differentiation which is typical of primary gastric cancer. This diagnostic pitfall is quite well documented in the literature [14-15].

In the clinical context, the distinction is also difficult if not impossible based on symptoms, radiology, and endoscopy images [16]. The clinical presentation of a gastric metastasis may be the same as a primary tumor appearing as a linitis plastica (also called water-bottle stomach), defined by a thickening mucosa with rigidity. However, approximately 50% of patients may have shallow mucosal lesions indistinguishable from benign gastric mucosal lesions [17]. In one series of Cormier’s study, all 31 patients with linitis plastica from metastatic breast cancer were found to have ILC [18].

When a diagnosis of ILC is made in core needle biopsy, it is advisable to perform a wide sampling of breast surgical specimens. This enables an accurate assessment of tumor size, which may be underestimated by macroscopic examination [19]. Despite a large sampling, the SRCC was not present in any sample taken by the breast resection.

Immunochemistry is required when a history of breast cancer is mentioned. GATA3 is a nuclear transcription factor, highly sensitive, expressed in >90% of breast carcinoma, but it is not specific because it is also expressed in urothelial carcinoma and squamous cell carcinoma [20]. Estrogen receptors and progesterone receptors are present in normal and neoplastic breast epithelium. Combining GATA3, ER, and PR contributed to the conclusion of a breast origin [20]. 

Our case is peculiar because the primary tumor showed no signet ring differentiation. This pattern appeared in the metastatic spread to the stomach thus making interpretation even more challenging. It was only with the full clinical history as well as the adequate immunohistochemical study that the correct origin of the observed signet ring cells was able to be determined.

 In order to explain these findings, and considering the lack of documented cases in the literature of the acquisition of an additional differentiation during the metastatic spread, we concluded that the signet ring cells component was most likely present focally in the primary breast tumor and thus not represented despite the wide sampling.

## Conclusions

In conclusion, we report a case of a recurrence of a classic invasive lobular carcinoma in the stomach appearing as a gastric SRCC, thus mimicking a primary poorly cohesive carcinoma. Clinical history knowledge and a pertinent immunochemistry panel are crucial to correctly assess the origin of the tumor and overcome this diagnostic pitfall. The distinction is necessary as the prognosis and approaches for treatment are different.
